# Synthesis, Characterization, and the Antioxidant Activity of Double Quaternized Chitosan Derivatives

**DOI:** 10.3390/molecules22030501

**Published:** 2017-03-22

**Authors:** Lijie Wei, Qing Li, Wenqiang Tan, Fang Dong, Fang Luan, Zhanyong Guo

**Affiliations:** 1 Key Laboratory of Coastal Biology and Bioresource Utilization, Yantai Institute of Coastal Zone Research, Chinese Academy of Sciences, Yantai 264003, China; ljwei@yic.ac.cn (L.W.); wqtan@yic.ac.cn (W.T.); fdong@yic.ac.cn (F.D.); fluan@yic.ac.cn (F.L.); 2 University of Chinese Academy of Sciences, Beijing 100049, China

**Keywords:** single quaternized chitosan, double quaternized chitosan, chemical modification, antioxidant activity

## Abstract

With the specialty of improving the water solubility of chitosan, quaternary ammonium salts have broadened the application of this polysaccharide in food, medicine and pesticides. To identify the effect of quaternary ammonium salts’ quantity, single quaternized chitosan *N*-phenmethyl-*N*,*N*-dimethyl chitosan (PDCS), double quaternized chitosan *N*-(1-pyridylmethyl-2-ylmethyl)-*N*,*N*-dimethyl chitosan (MP2MDCS), *N*-(1-pyridylmethyl-3-ylmethyl)-*N*,*N*-dimethyl chitosan (MP3MDCS), and *N*-(1-pyridylmethyl-4-ylmethyl)-*N*,*N*-dimethyl chitosan (MP4MDCS) were designed and synthesized successfully through chemical modification of chitosan. Besides, three kinds of antioxidant activities, including hydroxyl radicals, superoxide radicals, and 1,1-Diphenyl-2-picrylhydrazyl (DPPH) radicals were tested in vitro. As shown in this paper, the scavenging ability was ranking in order of MP3MDC > MP4MDCS > MP2MDCS > PDCS > chitosan at 1.6 mg/mL in all assays. All double quaternary ammonium salts were better than chitosan or the single quaternary ammonium salt. In addition, MP3MDCS could scavenge hydroxyl radicals totally at 1.6 mg/mL. MP2MDCS and MP4MDCS with more than 90% scavenging indices both had great scavenging ability on hydroxyl radicals or DPPH radicals. Furthermore, these data demonstrated that the increasing number of the positive charge would improve the antioxidant property of chitosan derivatives, and the *N*-pyridinium position would influence the scavenging radical ability.

## 1. Introduction

Free radicals, especially oxygen free radicals in one’s body, may damage the chemical structure of the organized cell and cause symptoms, such as ruptures of the main chain of the nucleic acid and protein peptide bond, membrane lipid peroxidation, enzyme inactivation, and cell apoptosis in certain pathological conditions [1,2,3,4,5]. Fortunately, free radicals can be removed by the antioxidant, where the composite agent plays an important role in cleaning while protecting body cells from damaging [6]. Meanwhile, it was reported that some polysaccharides with free hydroxyl and amino group have antioxidant ability, and the order of scavenging hydroxyl radicals ability is chitosan > hyaluronan > starch [7]. Chitosan and chitosan derivatives, in consequence, have attracted numerous attentions as the natural antioxidants with inestimable potentials [8].

Chitosan, the natural cationic amino polysaccharide copolymer of glucosamine and *N*-acetylglucosamine, is usually obtained from the exoskeletons of the shellfish and the insects. As a natural renewable resource, chitosan has advantages in unique physicochemical characteristics and bioactivities expressed as its antifungal, antioxidant and antitumor properties [9,10,11,12,13]. Due to the high degree of polymerization, however, this polysaccharide is limited by its poor solubility above pH 6.5 [14,15]. Therefore, functional derivatives prepared by chemical modification, such as quaternization, carboxylation, phosphorylation and sulfation, are introduced to enhance chitosan’s water solubility and bioactivity while sustaining its original biodegradability and biocompatibility [16,17,18,19,20,21]. Actually, with the fact that molecular structures determine the biological activities of polysaccharides, studies of the structure activity relationship of polysaccharides start to gain their popularity [22]. And, one branch of this promising research, the analysis of different molecular structure of quaternary ammonium salts and their antioxidant abilities are the main idea in following parts of this paper.

Pyridine was introduced into the polysaccharide backbone in this article, since pyridine was widely used in agrochemicals and pharmaceuticals with an aromatic heterocycle as an important solvent and reagent [23,24]. *N*-pyridylmethy chitosan was synthesized based on the reaction between the primary amino group of chitosan and aldehyde group of pyridine carboxaldehyde following by the reduction with sodium borohydride [25,26]. Besides, the pyridine was easily attacked by alkylating agents to obtain *N*-alkylpyridinium salt since its chemical property was similar to tertiary amine [24]. Besides, it was reported that pyridine chitosan derivatives could improve polysaccharide properties including solubility, physicochemical and biological properties, which could be applied in antimicrobial activity, sensor application, and biomedical application [27,28,29]. It was reported that pyridinium derivatives have been shown to be non-toxic for genen delivery in vitro [30]. Furthermore, *N*-pyridinium positions could influence the efficiency in mental adsorption, and gene carriers [30,31,32]. However, less attention has been paid to the influence of *N*-pyridinium position on the antioxidant activity. Therefore, double quaternary ammonium salts at different *N*-pyridinium position with a similar degree of substitution (DS) were investigated in this paper.

It was reported that quaternized chitosan derivatives had better antioxidant abilities than any of chitosan, Schiff bases of chitosan or *N*-substituted chitosan, and proposed that the antioxidant activity was affected by the positive charge of quaternized chitosan derivatives [33,34]. In that case, the density of positive charge could influence the efficiency of antioxidant activities. Therefore, in order to further study the influence of the positive charge and *N*-pyridinium position of quaternized chitosan derivatives on antioxidant activities, *N*-phenmethyl-*N,N*-dimethyl chitosan (PDCS), *N*-(1-pyridylmethyl-2-ylmethyl)-*N*,*N*-dimethyl chitosan (MP2MDCS), *N*-(1-pyridylmethyl-3-ylmethyl)-*N*,*N*-dimethyl chitosan (MP3MDCS), and *N*-(1-pyridylmethyl-4-ylmethyl)-*N*,*N*-dimethyl chitosan (MP4MDCS) were synthesized successfully via *N*-pyridylmethyl chitosan in this paper, and the antioxidant activity was also investigated systematically by the assessment of hydroxyl radicals’ scavenging activity, superoxide radicals’ scavenging activity, and DPPH radicals’ scavenging activity. In the meantime, FT-IR, ^1^H-NMR, and the elemental analyses characterized the chemical structure of the chitosan derivatives.

## 2. Results

### 2.1. Structure of the Chitosan Derivative

**Scheme 1:**
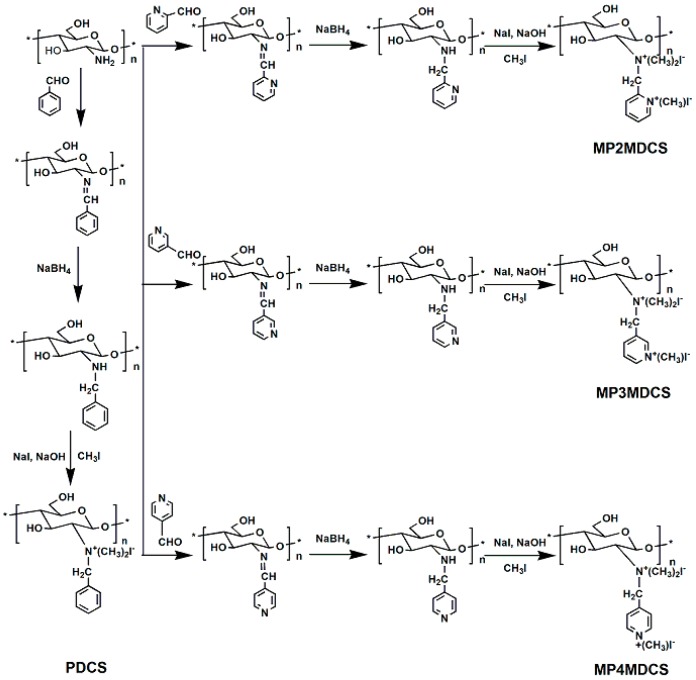
Synthetic pathway of PDCS, MP2MDCS, MP3MDCS, and MP4MDCS.

The synthetic procedures of the quaternary ammonium salts chitosan are shown in Scheme 1. *N*-(2-pyridylmethy), *N*-(3-pyridylmethy), and *N*-(4-pyridylmethy) chitosan were synthesized based on the reaction between the primary amino group of chitosan and aldehyde group of pyridine carboxaldehyde following by the reduction with sodium borohydride. Then, the secondary amine and *N*-pyridine were attacked by iodomethane to obtain quaternary ammonium salts, respectively.

**Figure 1:**
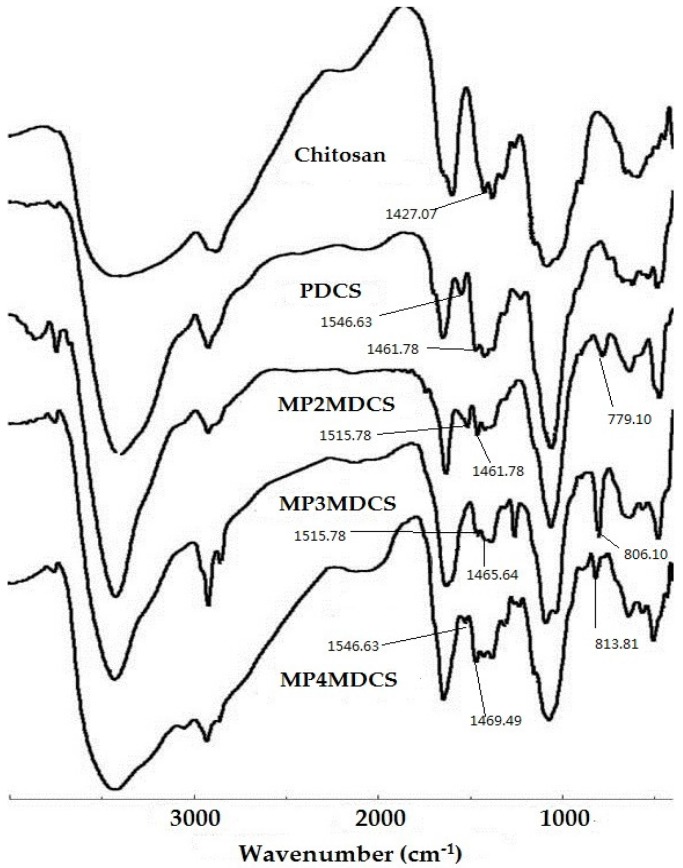
FT-IR spectra of Chitosan, PDCS, MP2MDCS, MP3MDCS, and MP4MDCS.

The FT-IR spectrum data of chitosan, PDCS, MP2MDCS, MP3MDCS, and MP4MDCS were shown in Figure 1. The spectrum of chitosan showed that saccharide mainly contained the following characteristic bands: ν (O-H) or ν (N-H) 3428.81 cm^−1^, ν (C-H) 2919.70 cm^−1^, ν (O=C-NH I band) 1643.05 cm^−1^, δ (C-H) 1427.07, 1380.78 cm^−1^, ν (O=C-NH III band) 1322.93 or 1261.22 cm^−1^, ν (C-O) 1068.37 cm^−1^, and 898.67 cm^−1^ indicated the β glycosidic bond. After quaternized, a new peak appeared at about 1546.63 cm^−1^ for PDCS, which was assigned to the benzene ring, and the peak at about 1461.78 cm^−1^ was the characteristic absorption of N-CH_3_ [35]. The peaks of quaternary ammonium salts of MP2MDCS, MP3MDCS, and MP4MDCS appeared at 1515.78 cm^−1^, 1515.78 cm^−1^, and 1546.63 cm^−1^, respectively. The absorption of N-CH_3_ was at about 1461.78 cm^−1^, 1465.64 cm^−1^, and 1469.49 cm^−1^ for MP2MDCS, MP3MDCS, and MP4MDCS, respectively. Moreover, double quaternized chitosan MP2MDCS, MP3MDCS, and MP4MDCS had new peaks at 779.10 cm^−1^, 806.10 cm^−1^, and 813.81 cm^−1^, respectively, corresponding to the pyridine groups with different substitution position. Above results demonstrated preliminarily that quaternized chitosan derivatives were obtained.

**Figure 2:**
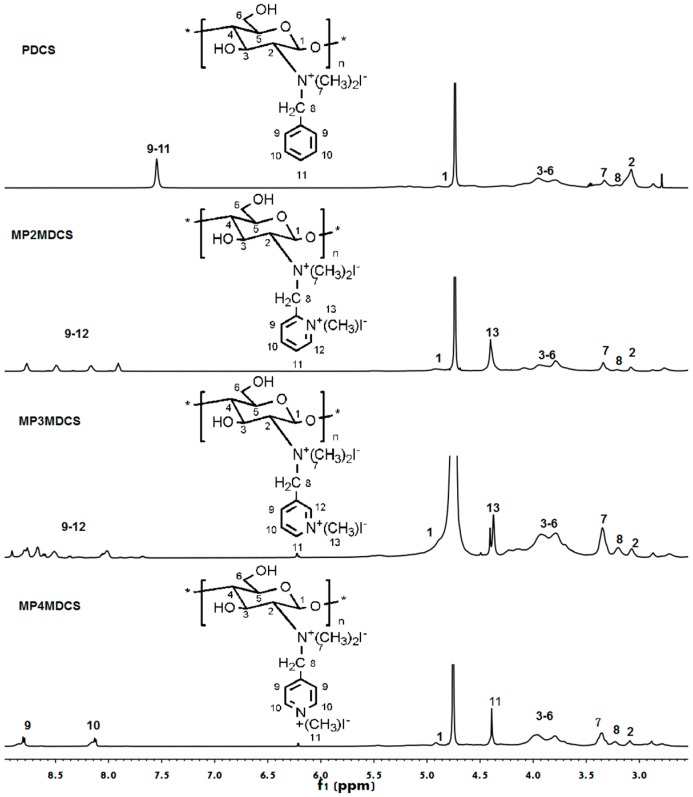
^1^H-NMR spectra of PDCS, MP2MDCS, MP3MDCS, and MP4MDCS.

Figure 2 showed the ^1^H-NMR spectra of PDCS, MP2MDCS, MP3MDCS, and MP4MDCS, respectively. It was known that all of the signals at 5.12 to 3.81 ppm were assigned to the protons of glucose skeleton of chitosan. It exhibited characteristic resonance of N-CH_3_ at about 3.35 ppm for C7 in the molecules of PDCS, MP2MDCS, MP3MDCS, and MP4MDCS, respectively. At the same time, the peaks at 4.42, 4.38, and 4.39 ppm should correspond to methyl protons grafted to pyridine for MP2MDCS, MP3MDCS, and MP4MDCS, respectively. And 8.0–9.3 ppm should correspond to the pyridine ring with different substitution position. The signal at 7.5 ppm was assigned to the benzene ring. The above mentioned results demonstrated further that PDCS, MP2MDCS, MP3MDCS and MP4MDCS were obtained successfully.

### 2.2. Antioxidant Activity

Chitosan has poor solubility in neutral water due to the high polymerization degree. We used the water-soluble chitosan with low molecular weight in all antioxidant activity tests. All quaternized chitosan derivatives had good solubility in water, and were prepared as aqueous solutions at the concentration of 0.1 to 1.6 mg/mL.

**Figure 3:**
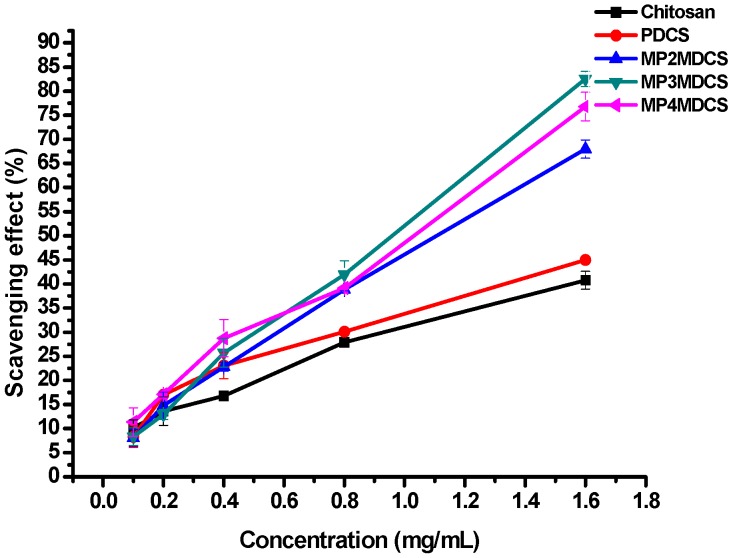
Superoxide radicals’ scavenging ability of Chitosan, PDCS, MP2MDCS, MP3MDCS and MP4MDCS.

Figure 3 showed the superoxide radicals’ scavenging ability of chitosan and all quaternized chitosan derivatives composed at 0.1 to 1.6 mg/mL. According to the graph, we concluded the results as follows: Firstly, the superoxide radicals’ scavenging ability of all samples enhanced with the increasing concentration. Secondly, scavenging indices were listed as follows at the concentration of 1.6 mg/mL: chitosan 40.75%, PDCS 43.04%, MP2MDCS 67.98%, MP3MDCS 82.53%, and MP4MDCS 76.80%. These data showed that MP2MDCS, MP3MDCS and MP4MDCS had better superoxide radicals’ scavenging ability than chitosan and PDCS at 1.6 mg/mL. And all double quaternized chitosan derivatives had higher density of positive charges than chitosan and PDCS, which might conclude that the higher density of positive charges could contribute to the scavenging on the superoxide radicals’ activity. Thirdly, in the three double quaternized chitosan derivatives, the scavenging properties of MP2MDCS, MP3MDCS, and MP4MDCS were similar at the lower concentration, but MP3MDCS gave much stronger scavenging ability at 1.6mg/mL, which might conclude that the different position of *N*-pyridinium might have some influence on the scavenging activity.

**Figure 4:**
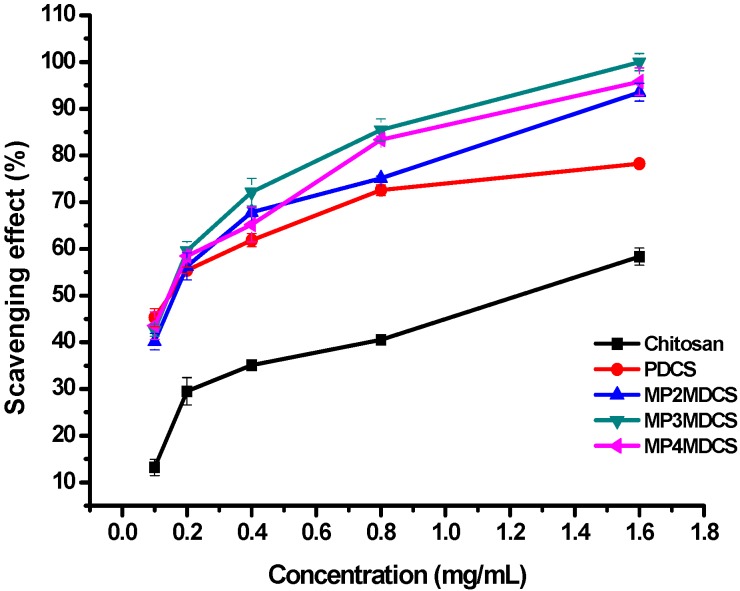
Hydroxyl radicals’ scavenging ability of Chitosan, PDCS, MP2MDCS, MP3MDCS and MP4MDCS.

Figure 4 showed the curve chart of the hydroxyl radicals’ scavenging ability of chitosan and the synthesized quaternized chitosan derivatives composed at 0.1 to 1.6 mg/mL. The results were similar to above results on the superoxide radicals’ scavenging activity. Firstly, the scavenging indices enhanced with the increasing concentration. Secondly, the scavenging ability against hydroxyl radicals was in order of MP3MDCS > MP4MDCS > MP2MDCS > PDCS > chitosan at the 1.6 mg/mL. Thirdly, MP3MDCS could scavenge hydroxyl radicals totally at 1.6 mg/mL.

**Figure 5:**
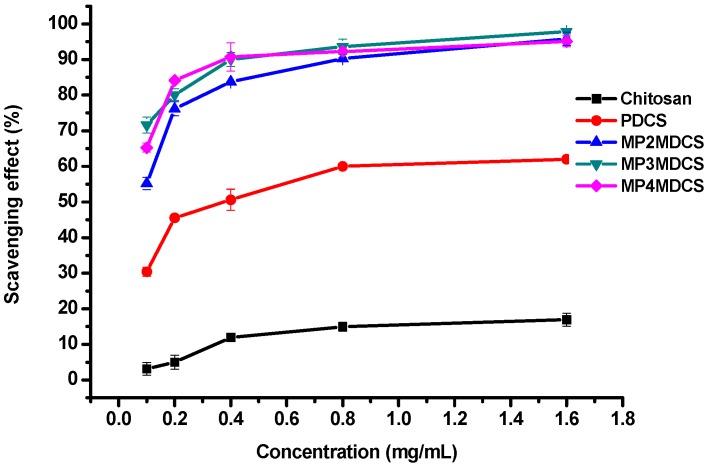
DPPH radicals scavenging ability of Chitosan, PDCS, MP2MDCS, MP3MDCS, and MP4MDCS.

The scavenging abilities of chitosan, PDCS, MP2MDCS, MP3MDCS, and MP4MDCS against DPPH radicals were shown in Figure 5. The results were similar to those of scavenging superoxide radicals and hydroxyl radicals too. Firstly, the sample had a positive correlation with the increasing concentration. Secondly, the scavenging indices were listed as followed: Chitosan 16.93%, PDCS 62.60%, MP2MDCS 94.80%, MP3MDCS 97.80%, and MP4MDCS 95.08%. All double quaternary ammonium salts could improve the ability of scavenging DPPH radicals significantly.

Based on the results mentioned above, the scavenging ability of the products against superoxide radicals, hydroxyl radicals, and DPPH radicals were almost in order of MP3MDCS > MP4MDCS > MP2MDCS > PDCS > Chitosan at 1.6 mg/mL, which could conclude that the antioxidant ability might associate with the density of the positive charge, as the positive charge could attract the single electron of free radicals to damage the free radical chain reaction. All double quaternized chitosan derivatives with higher density positive charges than chitosan and PDCS would attract more single electron of free radicals, which could improve the antioxidant ability. Furthermore, different *N*-pyridinium positions could have different influences on the antioxidant activity. The delocalization of pyridine was remarkable at the 2- and 4-position, which was enhanced if the nitrogen was protonated. So the distribution electronic cloud of MP2MDCS and MP4MDCS were more uniform than MP3MDCS in pyridine ring, which could explain MP3MDCS had a better antioxidant ability than MP2MDCS and MP4MDCS [30,36,37]. Based on the above results, it will be reasonable to presume that the density of positive charges and the different *N*-pyridinium position can influence the antioxidant property of chitosan derivatives. Further comprehensive investigation to ascertain the antioxidant mechanism and the structure–activity relationship would be studied in the future.

## 3. Materials and Methods

### 3.1. Materials

Chitosan (MW 2.0 × 10^5^, the degree of deacetylation 97%) was purchased from Qingdao Baicheng Biochemical Corp. (Qingdao, China). In addition, 2-pyridinecarboxaldehyde, 3-pyridinecarboxaldehyde and 4-pyridinecarboxaldehyde were purchased from Aladdin Industrial Corp. (Shanghai, China) Sodium borohydride (NaBH_4_), *N*-methyl-2-pyrrolidone (NMP), iodomethane (CH_3_I), sodium iodide (NaI), and sodium hydroxide (NaOH) were purchased from Sinopharm Chemical Reagent Co., Ltd. (Shanghai, China).

### 3.2. Analytical Methods

FT-IR spectrometers were recorded on a Jasco-4100 ranging from 4000 cm^−1^ to 400 cm^−1^ (Japan, provided by JASCO Co., Ltd., Shanghai, China) with KBr disks. ^1^H NMR was recorded on a Bruker AVIII 500 spectrometer (Fällanden, Switzerland, provided by Bruker Biospin CN/Bruker (Beijing, China) Tech. and Serv. Co., Ltd., Beijing, China), using D_2_O as solvents with tetramethylsilane (TMS) as internal standard. Chemical shift values were given in δ (ppm). The elemental analyses (C, H, and N) were performed on a Vario EL III (Elementar, Langenselbold, Germany). The Degree of Substitution (DS) of chitosan derivatives were calculated based on the percentages of carbon and nitrogen, which is acquired by following Fonseca et al.’s method [38]. The UV-vis absorbance of the tested mixture were measured with a T6 New Century UV spectrometer (China, provided by P General Co., Ltd., Beijing, China). The results are processed by computer programs Excel (Microsoft, Redmond, WA, DC, USA) and Origin 8 (OriginLab, Northampton, MA, USA) and reported as mean ± SD.

### 3.3. Synthesis of Single Quaternized Chitosan (PDCS)

Single quaternized chitosan PDCS was prepared according to an earlier method [34]. In brief, 1.61 g chitosan was dissolved into 50 mL 1% acetic acid aqua and 50 mL ethanol in flask at 25 °C, and 3.05 mL benzaldehyde were added with stirring at 25 °C. After 2 h, 1.8 g NaBH_4_ was added and the reaction was carried out for 2 h. The solution was precipitated into acetone and the precipitants were filtered. Then, the *N*-substituted chitosan derivative was obtained after drying at 60 °C for 24 h. Then, 0.5 g *N*-substituted chitosan was dispersed into 30 mL *N*-methyl-2-pyrrolidone (NMP) for 12 h at 25 °C. To this mixture, 0.1 mL NaOH (1 M), 0.75 g NaI, and 2 mL CH_3_I were added, and the reaction was refluxed gently with stirring at 60 °C for 4 h. The solution was precipitated by excess acetone and the precipitations were filtered. The single quaternized chitosan derivative was obtained by drying at 60 °C for 24 h (Scheme 1), yield: 90.30%; DS: 78.32% (Table 1).

### 3.4. Synthesis of Double Quaternized Chitosan (MP2MDCS, MP3MDCS, and MP4MDCS)

Double quaternized chitosan MP2MDCS, MP3MDCS, and MP4MDCS were synthesized as follows: 1.61 g chitosan was dissolved into 50 mL 1% acetic acid aqua and 50 mL ethanol in flask at 25 °C, and 30 mmol 2-pyridinecarboxaldehyde (2.85 mL), 3-pyridinecarboxaldehyde (2.82 mL), and 4-pyridinecarboxaldehyde (2.86 mL)were added, respectively, with stirring at 25 °C. After 2 h, 1.8 g NaBH_4_ was added and the reaction was carried out continuously for 2 h. The solution was precipitated into excess acetone and the precipitant were filtrated. Then, the *N*-methylpyridine chitosan derivatives were obtained after drying at 60 °C for 6 h. In addition, 0.5 g above synthesized *N*-methylpyridine chitosan was dispersed into 30 mL NMP for 12 h at 25 °C. The reaction was carried out at 60 °C for 4 h with reflux stirring after 0.2 mL NaOH solution (1 M), 1.5 g NaI and 4 mL CH_3_I were added. The solution was precipitated by excess acetone and the precipitations were filtered. The double quaternized chitosan derivatives were obtained by drying at 60 °C for 24 h (Scheme 1), MP2MDCS yield: 93.54%; DS: 88.0%; MP3MDCS yield: 94.62%; DS: 76.5%; MP4MDCS yield: 93.80%; DS: 77.0% (Table 1).

### 3.5. Hydroxyl Radicals’ Scavenging Activity Assay

The reaction of Fe-EDTA complex with H_2_O_2_ in phosphate buffer can generate ·OH, which is harmful to the body through reacting with biological molecule such as amino acid or DNA. The hydroxyl radical scavenging activity was measured according to Guo and Liu [5,34]. The reaction mixture, total volume 4.5 mL, containing the samples of chitosan or chitosan derivatives (10 mg/mL, 0.045, 0.09, 0.18, 0.36, and 0.72 mL), were incubated with EDTA–Fe^2+^ (220 µM), potassium phosphate buffer (150 mM, pH 7.4), safranine T (0.23 µM) and H_2_O_2_ (60 µM) for 30 min at 37 °C. The absorbance of the mixture was measured at 520 nm. Three replicates for each sample concentration were tested. The ·OH bleached the safranine T, so decreased absorbance of the reaction mixture indicated decreased ·OH scavenging ability, and the capability of scavenging ·OH was calculated using the follow equation: Scavenging effect (%) = (A_sample 520nm_− A_blank 520nm_)/(A_control 520nm_− A_blank 520nm_) × 100, where A_blank 520nm_ was the absorbance of the blank (distilled water instead of the samples), and A_control 520nm_ was the absorbance of the control (distilled water instead of H_2_O_2_).

### 3.6. Superoxide Radicals’ Scavenging Ability Assay

The superoxide radical ability was assessed by the method of Nishikimi et al. [39]. Superoxide radicals can generate single oxygen or hydroxyl radicals which could cause the peroxidation of lipids [40], which would be deleterious to the body. Involving testing samples of chitosan or chitosan derivatives (5 mg/mL, 0.06, 0.12, 0.24, 0.48, and 0.96 mL), 30 µM phenazine methosulfate (PMS), 338 µM nicotinamide adenine dinucleotide reduced (NADH), and 72 µM nitro blue tetrazolium (NBT) in Tris-HCl buffer (16 mM, pH 8.0), the reaction mixture was incubated at 25 °C for 5 min. The absorbance was read at 560 nm against a blank. Three replicates for each sample concentration were tested and the capability of scavenging superoxide radical was calculated using the following equation: Scavenging effect (%) = [1 − (A_sample 560nm_ − A_control 560nm_)/A_blank 560nm_] × 100, where A_control 560nm_ is the absorbance of the control (distilled water instead of NADH for each concentration) and A_blank 560nm_ is the absorbance of the blank (distilled water instead of the samples).

### 3.7. DPPH Radicals’ Scavenging Ability Assay

According to HU [35], the DPPH radical scavenging ability of chitosan, PDCS, MP2PDCS, MP3MDCS, and MP4MDCS were measured as followed: testing samples (10 mg/mL, 0.03, 0.06, 0.12, 0.24 and 0.48 mL) and 2 mL ethanol solution of DPPH (180 µmol/L) was incubated for 30 min at 25 °C. Then, the absorbance of the remained DPPH radical was measured at 517 nm against a blank. Three replicates for each sample concentration were tested and the scavenging effect was obtained according to the following equation: Scavenging effect (%) = [1 − (A_sample 517nm_ − A_control 517nm_)/A_blank 517nm_] × 100, where A_control 517nm_ is the absorbance of the control (ethanol instead of DPPH for each concentration) and A_blank 517nm_ is the absorbance of the blank (distilled water instead of the samples).

## 4. Conclusions

Via *N*-pyridylmethyl chitosan, a series of derivatives of chitosan with single or double quaternary ammonium salts were synthesized successfully. In addition, antioxidant activities of chitosan and quaternized chitosan derivatives against hydroxyl radicals, DPPH radicals, and superoxide radicals were tested in vitro. It was found that all quaternized chitosan derivatives had good water solubility and stronger antioxidant ability compared with chitosan, especially double quaternized chitosan derivatives that might be further developed into more effective antioxidant biomaterials. These data demonstrated that the higher positive charge density of quaternized chitosan derivatives might contribute to antioxidant activities. Furthermore, MP3MDCS was more effective than MP2MDCS and MP4MDCS in all assays especially at 1.6 mg/mL. It was reasonable to presume that the *N*-pyridinium position of double quaternized chitosan derivatives could influence the antioxidant property. Besides, it was reported that pyridinium derivatives were showed to be non-toxic for genen delivery in vitro among the quaternary ammonium chitosans [30], so our double quaternized chitosan derivatives might have lower toxicity, which needs to be studied further. Finally the mechanism of the antioxidant activity and the structure–activity relationship need to be further investigated in the future.

## Figures and Tables

**Table 1:** The elemental analyses, yields, and the degrees of substitution of chitosan derivatives.

Compounds	Yields (%)	Elemental Analyses (%)				Degrees of Substitution (%)	Deacetylation (%)
		C	N	H	C/N		
**Chitosan**		41.450	7.980	6.201	5.19		97
**PDCS**	90.30	34.553	3.072	5.465	11.24	78.9	
**MP2MDCS**	93.54	35.717	5.611	5.618	6.366	88.0	
**MP3MDCS**	94.62	31.645	5.026	5.373	6.296	76.5	
**MP4MDCS**	93.80	30.252	4.801	5.367	6.300	77.0	

## Abbreviations

The following abbreviation are used in this manuscript:

PDCS	*N*-phenmethyl-*N,N*-dimethyl chitosan
MP3MDCS	*N*-(1-methylpyridin-3-ylmethyl)-*N*,*N*-dimethyl chitosan
MP4MDCS	*N*-(1-methylpyridin-4-ylmethyl)-*N*,*N*-dimethyl chitosan
MP2MDCS	*N*-(1-methylpyridin-2-ylmethyl)-*N*,*N*-dimethyl chitosan
DPPH	1,1-Diphenyl-2-picrylhydrazyl
EDTA	Ethylenediaminetetraacetic acid
